# Notes and News

**Published:** 1898-02-26

**Authors:** 


### Notes and News.

(Collected and Communicated.)

HOSPITAL MEETINGS.

New Hospital for Women.—Annual Meeting, Thursday, March 3rd, 3.30, at the Hospital.

University College Hospital.—Annual Meeting, March 18th, 4 p.m., in the Board-room.

Great Northern Central Hospital.—Annual Meeting of Governors, Friday, February 25th, at 5 p.m., in the Board-room of the Hospital.

H.R.H. the Duke of Cambridge will preside at the Festival Dinner in aid of the North London Hospital for Consumption, to be held at the Hotel Cecil on May 19th.

Royal Hospital for Children and Women.—Annual Meeting, Monday, February 28th, at 3 o'clock, at the Mansion House.

H.R.H. the Duke of York has consented to become president of the National Society for the Employment of Epileptics.

The Worshipful Company of Goldsmiths have sent £1,000 to the London Hospital Maintenance Appeal Fund.

Two thousand pounds has just been presented to the Pasteur Institute in Paris by Madame Durand, who has expressed a wish that this sum should be devoted to the study of tuberculosis.

The *Laryngoscope* is now published simultaneously in Great Britain and America. The European editor is Dr. St. Clair Thomson, and the English publishers are Messrs. J. Wright and Co., of Bristol. The paper, as issued under the new arrangement, is a bright and interesting journal.

A correspondence has lately been going on in the *British Medical Journal* anent the old and many times threshed out subject of consultation with homœopaths. About the arguments used we have nothing to say just now, but we do enter a gentle protest against the use of the word "allopath" to indicate one who practises rational medicine. If it were used by one's enemy as a slander or a term of reprobation one would not mind, its obvious untruth would leave it harmless; but used by a friend, it just contains that vestige of truth which renders it offensive, for no doubt there are some, even of those who consider themselves orthodox of the orthodox, who attack symptoms by opposites with the same delightful indifference to pathology as is to be seen among those who affect the use of similars.

Owing to the epidemic of small-pox at Middlesbrough the North-Eastern Railway has discontinued running excursion trains from that town.

Mr. Noble Smith will lecture on "The Use of Apparatus in Orthopædic Surgery" at the City Orthopædic Hospital on Tuesday, March 1st, at five p.m.

In the Parliamentary Bill brought in by Sir Howard Vincent to restrain the immigration of aliens, he proposes to exclude from British ports "persons suffering from dangerous, contagious, or infectious disease."

Owing to the prevalence of measles on the battleships *Prince George* and *Repulse* these vessels have been ordered to leave the Channel Fleet and to proceed to Gibraltar, where the patients will be landed and the vessels fumigated.

It is satisfactory to find that the medical school in connection with the Cambridge University is likely to be better housed than has hitherto been the case. The site for a new building has been assigned by the Senate, and an appeal has been issued by Professor Allbutt for funds for the erection of a properly-fitted school of medicine. A sum of £20,000 will be required. Some of this has been already promised, the Mercers' Company having given a donation of £1,050, while handsome subscriptions are announced from Professor Allbutt himself, from members of the family of the late Professor Humphry, and from others. A meeting to promote the movement will be held in London on March 2nd, when it is hoped things will be placed on a satisfactory footing.

The weekly return of births and deaths issued by authority of the Registrar-General gives the death-rate in London during the past week as 21·5 per 1,000, against a death-rate of 20·4 per 1,000 in the 33 great towns. The total number of the deaths in London during the week was 1,856, of which 100 were from measles, 9 from scarlet fever, 33 from diphtheria, 53 from whooping-cough, 8 from enteric fever, and 8 from diarrhœa and dysentery. Thus 211 deaths were referred to these diseases, being 25 above the corrected average. The rise was by far the greatest in measles, which was 65 per cent. above the corrected average for the corresponding week. The deaths attributed directly to influenza numbered 87, having been 88, 102, and 98 in the preceding three weeks.

The following gentlemen have been selected as house officers at St. Thomas's Hospital from Tuesday, March 1st, 1898 (subject to the approval of the treasurer). House Physicians: H. E. Hewitt, H. H. Scott, H. F. Shea, and H. C. Haslam. House Surgeons: J. F. McClean, H. J. Marriage, J. S. Hall, and H. H. Sanguinetti. Assistant House Surgeons: E. H. Cobb, A. C. Robinson, F. L. A. Greaves, and A. H. Greg. Obstetric House Physicians: (Senior) S. D. Turner and (Junior) H. T. M. Alford. Ophthalmic House Surgeons: (Senior) F. A. C. Tyrrell and (Junior) S. N. Babington. Clinical Assistants: Special Department for Diseases of the Throat, A. J. Grant and A. F. Millar; Special Department for Diseases of the Skin, A. A. Osborne and A. C. Parsons; Special Department for Diseases of the Ear, F. R. Martin and C. A. Reynolds; Electrical Department, A. H. Gibbon.

